# Individual freedom versus collective responsibility: too many rights make a wrong?

**DOI:** 10.1186/1742-7622-3-14

**Published:** 2006-10-02

**Authors:** Katharine J Looker, Timothy B Hallett

**Affiliations:** 1 Department of Infectious Disease Epidemiology, Imperial College London, UK

## Abstract

Individuals might reasonably expect the freedom to make their own decisions regarding their health. However, what happens when an individual's wishes conflict with what is in that individual's best interests? How far should an individual's rights be restricted for his or her own benefit? Similarly, what limitations should be placed on an individual's behaviour when that person's wishes go against what is good for the population in general? Here we discuss the issues that can arise when the rights of individuals conflict with individual and population benefits in relation to infectious diseases.

When it comes to health, one might expect to have the freedom to behave as one chooses within the necessary legal constraints. An individual can decide, for instance, whether or not to seek treatment for a bacterial sexually transmitted infection (STI). The individual probably wants to be treated as this will alleviate her symptoms and minimise the possibility of associated complications such as infertility. With treatment, all her future sexual partners (and their partners, and so on) will also benefit since they will be at less risk of contracting the STI, and the burden on the healthcare system will consequently be reduced. This is a 'win-win' scenario; the individual wants treatment and this wish will benefit both that individual and the rest of the population. But what happens when the wants of the individual are not in line with what will benefit either the individual concerned or the general population? When these interests compete, what should be done?

We can imagine a range of scenarios where these tensions (individual-level wants versus individual-level benefits and individual-level benefits versus population-level benefits) are felt (Table 1). For example, an individual may want to keep a stockpile of the anti-influenza drug *Tamiflu*^® ^for his own personal use in case there is an epidemic, but this will not benefit the population if it deprives those most at need [1]. Or a parent, believing (incorrectly) that the measles-mumps-rubella (MMR) vaccine is linked to autism, may decline vaccination for her child despite this putting both the child and other individuals at risk of infection [2].

**Table 1:** A summary of potential conflicts between individual-level wants, individual-level health benefits and population-level health benefits.

**Individual wants?**	**Individual benefit exceeds individual harm?**	**Population benefit exceeds population harm?**	**Examples**
			Seeking treatment for a bacterial STD; staying home when ill with a gastro-intestinal infection
			Disclosing HIV status to partner; voluntarily revealing personal information for the purposes of disease surveillance
			Acquiring a personal stock-pile of *Tamiflu*^®^; being prescribed anti-malarials associated with least drug resistance
			Refusing the MMR vaccine; pressuring physician for antibiotics for a non-bacterial infection

			Forcing infected individuals to undergo treatment for TB; restricting the movement of persons during the SARS outbreak
			Forcibly quarantining suspected SARS cases; potentially rationing *Tamiflu*^® ^during an outbreak due to limited drug availability
			Withholding suppressive therapy which could otherwise prevent onward transmission of genital herpes because of contraindications in patient
			Withdrawing provision of antiretroviral therapy for HIV

On the other side of the coin are conflicts involving actions which an individual would not chose for himself but which nevertheless are good for the population. In the best case these actions benefit the individual too; for instance, an individual may not want to learn that he is infected with HIV, but knowing his HIV status will likely enable him to receive treatment, and might prevent him from transmitting the infection to others. Similarly, restricting movement during the Severe Acute Respiratory Syndrome (SARS) epidemic in 2003 reduced the risk of infection for everyone [3], though at the cost of some inconvenience to each individual. However, in the worst case a particular action does not only go against an individual's wishes but actually causes harm to that individual. For example, some uninfected individuals with flu-like symptoms could have been put at increased risk of infection with SARS if they were forcibly held in quarantine during the outbreak [4]. Of course, these issues will extend to even larger scales, pitting the benefits of different populations, countries or even regions against one another – for instance, would national stockpiles of *Tamiflu*^® ^be shared internationally in the face of a worldwide epidemic, or should each country seek to protect only its own people directly?

In which of these instances should collective benefit be prioritised even where this means curtailing individual freedom? What consideration should be given to what an individual 'wants' as opposed to what is good for her or good for everyone else? Economists, epidemiologists and policy-makers may find themselves naturally considering the population and maximising what is best overall, minimising loss of life, maximising benefit per cost and so on. But from an ethical or philosophical standpoint, that misses the importance of preserving individual freedom. One approach could be to consider individual-level wants, benefits and population-level outcomes at once, assigning a weighting to each. However, it is far from clear what the weightings would be, who would assign them, under which value system they would be assigned, and whether that would be comparing like-with-like. Individual-level benefits are often tangible, direct and characterised by a degree of certainty, whereas population benefits can be diffuse, come via circuitous unseen pathways and their eventual value may be less certain. Lastly, it is important to bear in mind that for even the most refined protocols, doctors only actually see individuals, and when listening to their personal concerns and wishes, population-level benefits – perhaps unproven, theoretical and anonymous – can seem rather distant.

These issues are not just abstract and in some cases have been tested in court. In 1905 the US Supreme Court for the first time upheld the right of the state to vaccinate everyone, even against their wishes [5]. A British man was convicted in 2005 of causing grievous bodily harm to a woman whom he knowingly placed at risk of contracting HIV [6]. Perhaps individuals would come to want what would actually benefit them if they appreciated the science behind the medicine, while the population-benefit could gain currency for the individual (and his or her doctor) through an awareness of the theoretical epidemiological arguments. But could individuals consistently want what is good for the population before themselves? Is this another example of when the 'good-for-the-group' instinct [7] is overridden by the compulsion for self-preservation [8]?

To offer their insights into these issues, we have invited members of inter-related disciplines to write briefly in reply to the question; "Under what circumstances should an individual's rights be restricted for the benefit of the individual or the population?" [9-11].
